# Myoferlin Depletion in Breast Cancer Cells Promotes Mesenchymal to Epithelial Shape Change and Stalls Invasion

**DOI:** 10.1371/journal.pone.0039766

**Published:** 2012-06-27

**Authors:** Ruth Li, William E. Ackerman, Cosmin Mihai, Leonithas I. Volakis, Samir Ghadiali, Douglas A. Kniss

**Affiliations:** 1 Laboratory of Perinatal Research, Division of Maternal-Fetal Medicine, Department of Obstetrics and Gynecology, The Ohio State University, Columbus, Ohio, United States of America; 2 Department of Biomedical Engineering, The Ohio State University, Columbus, Ohio, United States of America; 3 Division of Pulmonary, Allergy, Critical Care and Sleep Medicine, Department of Internal Medicine, The Ohio State University, Columbus, Ohio, United States of America; 4 Pacific Northwest National Laboratory, Richland, Washington, United States of America; Institute of Molecular and Cell Biology, Singapore

## Abstract

Myoferlin (MYOF) is a mammalian ferlin protein with homology to ancestral Fer-1, a nematode protein that regulates spermatic membrane fusion, which underlies the amoeboid-like movements of its sperm. Studies in muscle and endothelial cells have reported on the role of myoferlin in membrane repair, endocytosis, myoblast fusion, and the proper expression of various plasma membrane receptors. In this study, using an *in vitro* human breast cancer cell model, we demonstrate that myoferlin is abundantly expressed in invasive breast tumor cells. Depletion of MYOF using lentiviral-driven shRNA expression revealed that MDA-MB-231 cells reverted to an epithelial morphology, suggesting at least some features of mesenchymal to epithelial transition (MET). These observations were confirmed by the down-regulation of some mesenchymal cell markers (e.g., fibronectin and vimentin) and coordinate up-regulation of the E-cadherin epithelial marker. Cell invasion assays using Boyden chambers showed that loss of MYOF led to a significant diminution in invasion through Matrigel or type I collagen, while cell migration was unaffected. PCR array and screening of serum-free culture supernatants from shRNA^MYOF^ transduced MDA-MB-231 cells indicated a significant reduction in the steady-state levels of several matrix metalloproteinases. These data when considered *in toto* suggest a novel role of MYOF in breast tumor cell invasion and a potential reversion to an epithelial phenotype upon loss of MYOF.

## Introduction

Breast cancer is the second leading cause of cancer mortality in women [1], with the majority of the deaths due to metastatic rather than localized disease [2]. Unrestrained cell division elicited by somatic and/or germline mutations in several oncogenes and tumor suppressor genes such as *TP53* and *Rb,* and resistance to programmed cell death are hallmarks of tumorigenesis [3], [4]. For tumor cells to efficiently metastasize, they often undergo a pernicious transformation characterized by dramatically increased migration and invasive capacity. Specifically, the spread of cancer from a localized, self-contained tumor through tissue stroma and into distant organs requires that cells achieve atypical, robust motility and the capacity to aggressively degrade extracellular matrix (ECM), enabling them to invade surrounding tissues and vessels of the blood and lymphatic systems, and subsequently establish nascent, secondary tumors [5], [6].

The enhanced migration and invasive capacity of metastatic tumor cells are the subject of intense investigation, and it is now appreciated that several forms of cancer cell motility exist (i.e., single-cell, mesenchymal and amoeboid, protease-dependent, protease-independent, and collective migration) [7], [8]. Considerable progress has been made in identifying the potential molecular components that mediate cell migration, and there is growing evidence that intracellular vesicle trafficking of key proteins is crucial for efficient migration [9]. In tumor cell migration, the recycling of focal adhesion proteins (i.e., integrin receptor recycling) through endocytosis/exocytosis is now thought to contribute to the maintenance of polarized movement [10], [11]. Moreover, endocytosis/exocytic trafficking has been implicated in the delivery of proteolytic enzymes - including matrix metalloproteinases (MMPs) - to invadopodia, specialized protrusions utilized by cells for degradation of ECM [12], [13].

Ferlin proteins, an evolutionarily ancient family of large integral membrane proteins [14], have been implicated in vesicle trafficking in a variety of physiological settings. All mammalian ferlins derive their names based on homology to the *C. elegans* protein FER-1 (FERtilization defective-1). In roundworms, *fer-1* is required for the fusion of specialized vesicles (membranous organelles) with the plasma membrane at the leading edge of cell migration in spermatozoa; in the absence of functional FER-1 protein, the normal amoeboid locomotion of sperm is impaired, and infertility results [15]. Human patients harboring FER1L1 (FER-1-like 1, dysferlin) mutations manifest one of two autosomal recessive forms of muscular dystrophy - limb girdle muscular dystrophy type 2B and Myoshi myopathy [16], due to the inability of skeletal muscle fiber sarcolemma to repair damaged muscle cells during the normal course of biomechanical wear and tear [17]–[19]. Mutations in FER1L2 (otoferlin) result in non-syndromic deafness (DFNB9), due to the failure of synaptic vesicles to fuse and exocytose their cargo at the presynaptic plasma membrane [20], [21]. FER1L3 (myoferlin, MYOF), historically considered to be a muscle-specific protein, has not yet been directly associated with a distinct mammalian disorder, but recent studies have indicated that its deletion results in impaired mouse myoblast fusion into mature skeletal myotubes [22]. Furthermore, MYOF has been shown to mediate caveolae-dependent endocytosis in human endothelial cells [23].

Recent reports indicate that MYOF is critically involved in the function and/or stability of plasma membrane receptor tyrosine kinases (RTKs). Bernatchez *et* *al.* demonstrated that ablation of MYOF in vascular endothelial cells resulted in instability and rapid degradation of the vascular endothelial growth factor receptor 2 (VEGFR-2) [24]. Further research demonstrated that the expression of another angiogenic tyrosine kinase receptor, Tie-2, is also attenuated upon MYOF depletion in endothelial cells [25]. Finally, Demonbreun *et* *al.* reported that knockout of MYOF in mouse muscle resulted in diminished insulin-growth factor-1 receptor (IGFR) response and accumulation of the receptors in vesicles targeted for degradation [26].

The studies reported above when considered *in toto* suggest that MYOF may be a regulator of vesicle fusion events that deliver essential cargos (e.g., growth factor receptors, cell adhesion molecules, and other cell-surface proteins) to and/or from the plasma membrane. Inasmuch as tumor cell metastatic dissemination involves the convergence of receptors, signal transduction pathways, adhesion and matrix proteolytic molecules at the leading edge of a tumor cell, we proposed that MYOF (and perhaps other ferlin proteins) may be involved in one or more steps in tumor progression and/or spread. Microarray and proteomic studies in the cancer literature have reported MYOF expression in breast cancer specimens and relevant cell lines [27]–[29]. For example, in one microarray study, MYOF was 1 of 39 genes found to be over-represented in breast carcinoma [28]. These observations prompted us to hypothesize that MYOF may be an important protein in breast cancer cells for their mobilization during cellular migration and/or invasion.

We recently reported on the mathematical modeling of the role of MYOF in breast cancer cell invasion [30]. In the current manuscript, we present novel data confirming the computational modeling and show, in addition, that MYOF-deficient MDA-MB-231 human breast cancer cells exhibit a more epithelial shape compared to the mesenchymal morphology of wild-type MDA-MB-231 cells, suggesting that some features of a mesenchymal-to-epithelial transition (MET) phenotype [31] may exist in the absence of functional MYOF protein. Matrigel and collagen I based invasion assays demonstrated that depletion of MYOF diminished the invasive ability of cancer cells. In congruence with the mathematical modeling results, we report that the decreased invasive capacity is due, at least in part, to the down-regulation of MMP expression in MYOF-deficient cells.

## Results

### Myoferlin in Breast Cancer Cells Correlates with Cell Invasiveness

To correlate the expression of MYOF with the invasive capacity of breast cancer cells, we analyzed five mammary cell lines: MCF-10A, MCF-7, T47D, BT549, and MBA-MB-231. The MCF-10A line was chosen as a model for non-cancerous, non-invasive mammary epithelial [32]. MCF-7 and T47D cells model non−/low-invasive breast cancers, while BT549 and MDA-MB-231 have high invasive capacity [33].

**Figure 1:**
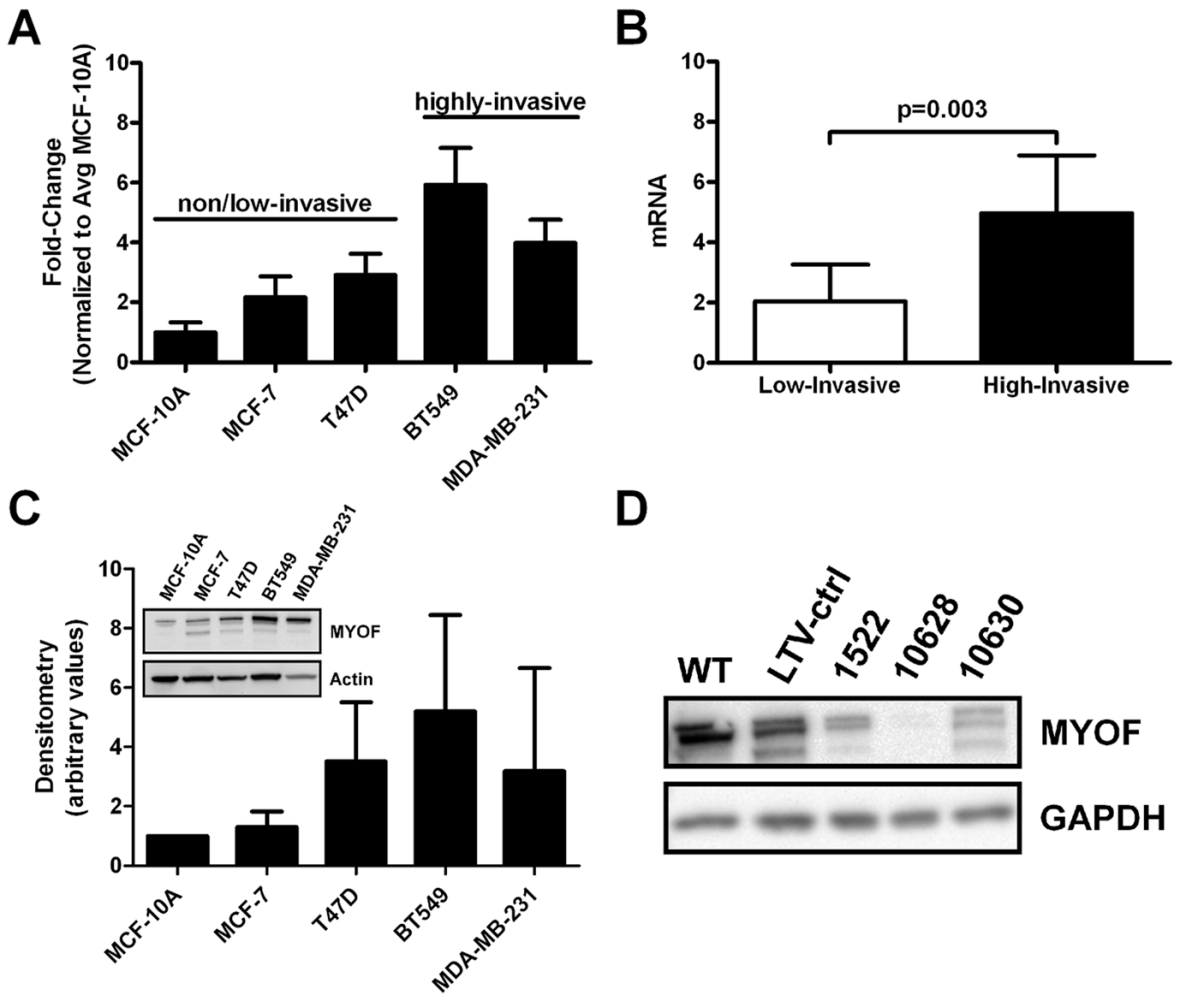
Myoferlin expression in breast cancer. (A) Quantitative RT-PCR results of MYOF mRNA levels in a panel of breast cancer cell lines, normalized to 18 S and compared to the nonmalignant breast epithelial cell line MCF-10A (mean ± s.d., *n* = 3 per cell type). No statistical significance was detected in comparing pairs of cell lines (Kruskal-Wallis with Dunn's multiple comparison test). (B) Representation of the data in subpanel A grouped by known invasive capacity of the cells (2 tailed, *p* = 0.003, Mann Whitney). (C) Immunoblotting for MYOF in breast cancer cell lines. Densitometry measurements was done by normalizing the density of MYOF to respective GAPDH or actin staining, and further normalized to the nonmalignant mammary epithelial cell line MCF-10A (*n* = 4, no statistical significance detected by Kruskal-Wallis with Dunn’s multiple comparison test). An immunoblot image is shown in the subpanel. (D) Myoferlin knockdown in breast cancer cells. Immunoblot showing the expression of MYOF in MDA-MB-231 wild type (WT), lentiviral transduction control (LTV-ctrl), and MYOF knockdown cell lysates using three Sigma-Aldrich MISSION® shRNA constructs (#1522, 10628, and 10630).

Examination of MYOF mRNA expression in each of the cell lines indicated that MYOF expression was 2.44-fold higher in the invasive lines (BT549 and MDA-MB-231) relative to non−/low-invasive cells (MCF-10A, MCF-7, and T47D) (Figure 1A and B). In congruence, higher MYOF protein expression was observed by immunoblotting in the highly-invasive breast cancer cell lines relative to those with low invasive potential (Figure 1C). To expand upon this, we examined the transcriptional profiling data available on 51 established breast cancer lines generated by Neve and colleagues (http://cancer.lbl.gov/breastcancer/data) [34]. Those 14 cell lines which clustered into the invasive, basal B-like subset exhibited statistically significantly greater MYOF expression compared to the 25 cell lines of the luminal phenotype as determined by two MYOF probesets (201798_s_at and 211864_s_at, Figure S1).

Building on these observations, we mined published microarray data available through the ArrayExpress database (www.ebi.ac.uk/arrayexpress) [35]. Our query returned microarray data (accession: E-TABM-276) from Cheng *et* *al.* [36] consisting of a panel of 23 microdissected primary invasive ductal carcinoma tissues, 28 adjacent stromal tissues, and 10 healthy controls. When MYOF expression was categorized by disease state, we observed significantly higher MYOF mRNA expression in tissues from patients with invasive ductal carcinoma compared with samples from healthy individuals (Figure S2). Collectively, these data suggest a potential correlation between MYOF over-expression and invasive breast carcinoma.

### Myoferlin Knockdown Promotes a Mesenchymal to Epithelial Change

Given a possible association between MYOF expression and invasive potential, we examined whether MYOF contributed to breast cancer cell invasion. We selected the MDA-MB-231 cells for these experiments since they exhibited high levels of MYOF protein expression in our survey, and were previously shown to have the greatest invasive capacity compared to a large panel of breast cancer cell lines [34]. Stable lines of MYOF-deficient MDA-MB-231 cells (231^MYOF-KD^) were generated using lentivirus-based delivery of short hairpin ribonucleic acids (shRNAs) targeting human MYOF (Figure S3). Control cell lines (231^LTV-ctrl^) were generated in parallel using a non-human gene targeting construct. Selective knockdown of MYOF protein expression was confirmed by immunoblotting (Figure 1D). Of the three constructs initially screened (#10628, 10630, and 1522), #10628 yielded the most efficient knockdown (mean 94% compared with controls, *n* = 4) and was used in subsequent experiments.

**Figure 2:**
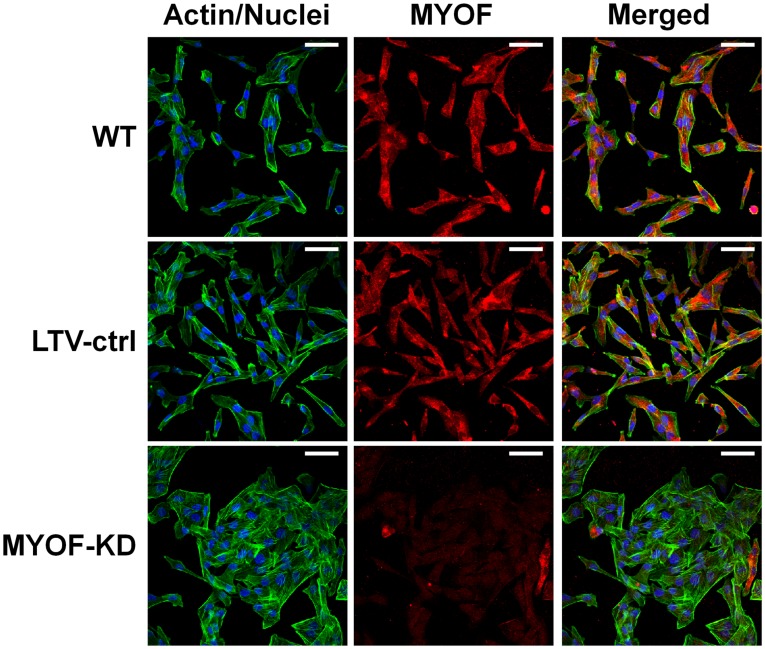
Morphology change following myoferlin depletion in MDA-MB-231 cells. Immunofluorescence micrographs showing the morphology of MDA-MB-231 wild type (WT), lentiviral transduction control (LTV-ctrl), and myoferlin knockdown (MYOF-KD) stable cell lines in culture. Note the more epithelial morphology of MYOF-KD cells compared to the more mesenchymal appearance of the WT and LTV-ctrl cells. Bar = 50 µm.

**Figure 3:**
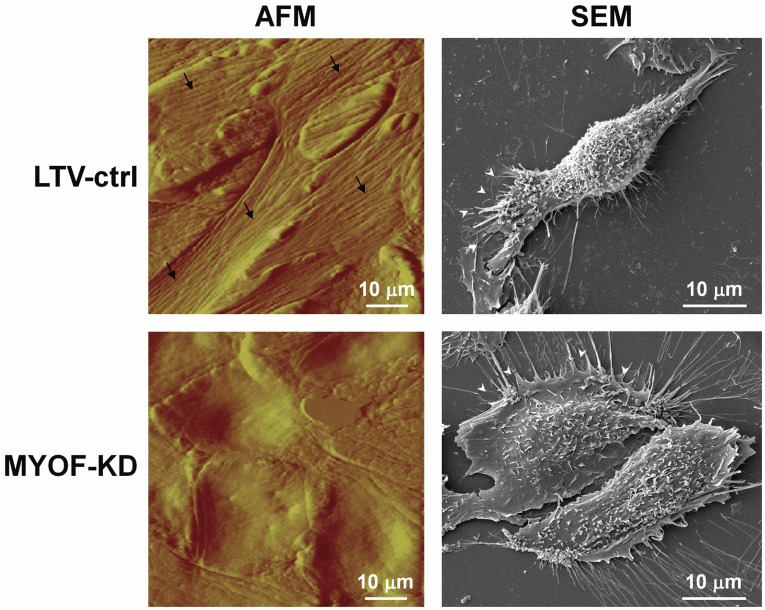
Morphology change following myoferlin depletion in MDA-MB-231 cells. Atomic force and scanning electron microscopy images showing the spindle, elongated shape of lentiviral control (LTV-ctrl) cells and the more flat and circular morphology of myoferlin depleted (MYOF-KD) cells. AFM imaging shows pronounced actin stress fibers (black arrows) oriented along the long axis being evident in the control but not in the MYOF depleted cells. Cytoplasmic poles, lamellipodia and filopodia are observable in the SEM images. White arrowheads indicate the leading edge of cells.

An unexpected, but interesting shape change appeared in the 231^MYOF-KD^ cells, wherein they exhibited a more epithelial-like (cobblestone) morphology which diverged from the fibroblastic, spindle shape of the wild type and 231^LTV-ctrl^ cells (Figures 2 and 3). The 231^MYOF-KD^ morphology was stable, and was maintained after 10 continual passages in culture (data not shown). By immunofluorescent microscopy, we noted that 231^MYOF-KD^ cells formed more cell clusters relative to control cells. When examined in greater detail using atomic force and scanning electron microscopy, the morphological divergence between 231^MYOF-KD^ and control cells was very prominent (Figure 3). At the cellular level, 231^LTV-ctrl^ cells often appeared elongated with tapering cytoplasmic poles, having one or more small lamellipodia and relatively short filopodia, and actin filaments oriented along the long axis of the cell (Figure 3). In contrast, 231^MYOF-KD^ cells exhibited a flattened, polygonal shape with broad lamellipodia, and elongated filopodia that tended to orient toward neighboring cells. Collectively, these results suggested a potential reversion of epithelial-mesenchymal transition, i.e., mesenchymal-epithelial transition (MET), at the morphology level.

**Figure 4:**
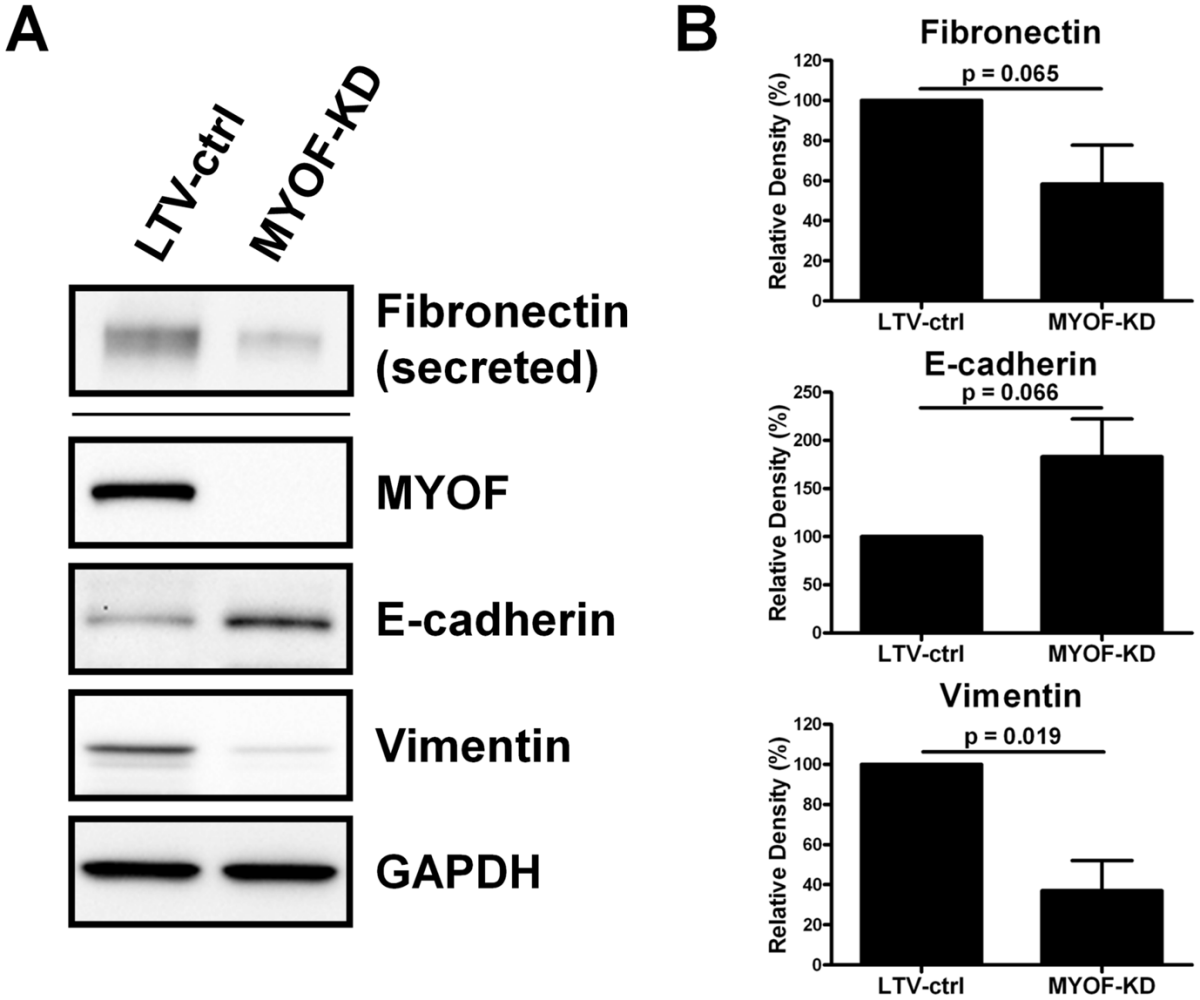
Expression of EMT markers following myoferlin depletion in MDA-MB-231 cells. (A) Representative immunoblots (*n* = 3) of select EMT markers in lentiviral control (LTV-ctrl) and myoferlin depleted (MYOF-KD) cells serum-starved for 24 h. (B) Graphs illustrating the semi-quantitative evaluation of the expression of EMT markers by densitometry analysis of blots (*n* = 3). Density was presented in the graph as “relative density (%)” with density of lentiviral controls normalized to 100%, and statistical testing was done with the 1-sample t-test.

The MET-like morphological changes prompted us to examine whether the expression of common epithelial-mesenchymal transition (EMT) markers (e.g. vimentin, fibronection, N-cadherin, and E-cadherin) was affected by MYOF depletion. By immunoblotting, 231^MYOF-KD^ cells expressed higher levels of the epithelial marked E-cadherin, and lower levels of mesenchymal markers, fibronectin and vimentin, than 231^LTV-ctrl^ cells (Figure 4). MDA-MB-231 cells do not natively express N-cadherin, so it was not surprising that N-cadherin was undetectable in 231^LTV-ctrl^ and 231^MYOF-KD^ cells (data not shown). The results of this screen of EMT markers suggest that the morphologic shift associated with MYOF-depletion may be correlated with MET changes at the molecular level. In a survey using a qRT-PCR-based array of EMT markers, we found that the mRNA levels appeared to correlate with the Western results in that fibronection mRNA was down-regulated 3.46-fold while that of E-cadherin was up-regulated 2.58-fold in 231^MYOF-KD^ cells compared with controls, although no significant change was found for vimentin (Table S1).

### Myoferlin Depletion Does Not Alter Cell Proliferation or Cell Cycle Distribution

We next subjected 231^LTV-ctrl^ and 231^MYOF-KD^ cells to proliferation assays. First, to assess general proliferative status, MTS assays were conducted on wild-type, 231^LTV-ctrl^, and 231^MYOF-KD^ cells. We observed no change in the growth rates of the three cell types (Figure S4A), indicating that MYOF does not profoundly alter tumor cell growth. To verify this result, we then carried out cell cycle analysis on the three cell types. Again, we detected no change in cell cycle activity in MYOF-depleted cells compared to wild-type or 231^LTV-ctrl^ cells (Figure S4B).

### Myoferlin Depletion Attenuates Cell Invasiveness

**Figure 5:**
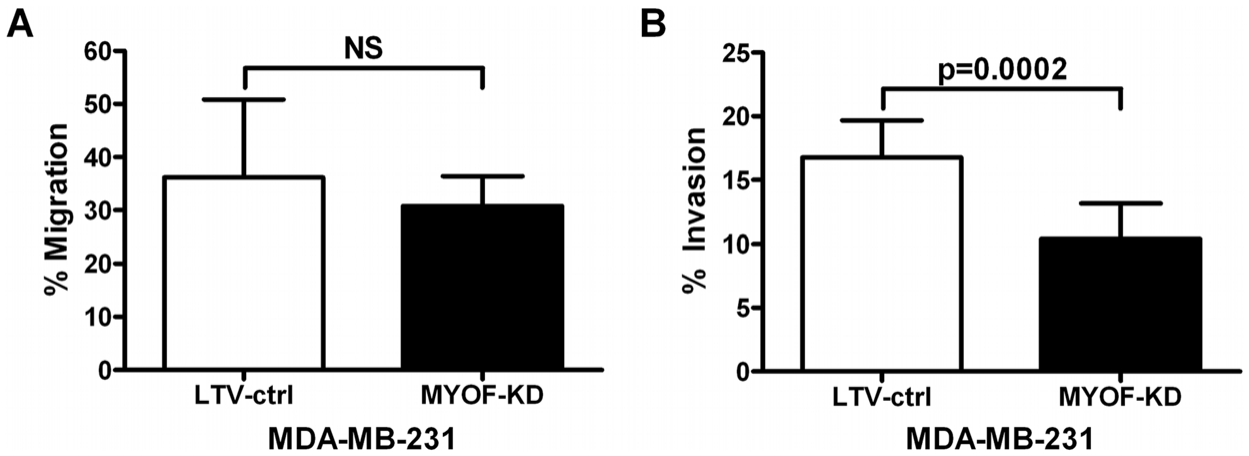
Myoferlin depletion reduces invasive but not migratory capacity of MDA-MB-231s control (LTV-ctrl) and MYOF depleted cells (MYOF-KD). (A) Boyden chamber migration assay of MDA-MB-231 cells moving across 8 µm porous membranes towards a 10% serum gradient for 24 h (mean ± s.d., *n* = 3, unpaired 2-tailed t-test). (B) 24 h Boyden chamber invasion results of MDA-MB-231 cells across a 100% Matrigel coated 8 µm porous membrane towards a 10% serum chemoattractant (mean ± s.d., *n* = 3, unpaired 2-tailed t-test).

MYOF deficiency was not associated with a change in chemotaxis (Figure 5A) in Boyden chamber assays when 10% fetal bovine serum was used as the chemoattractant. However, 231^MYOF-KD^ cells exhibited a significant ∼38% reduction in invasiveness into Matrigel relative to 231^LTV-ctrl^ cells (Figure 5B). These results suggest that targeted disruption of MYOF expression selectively attenuates invasiveness through reconstituted basement membrane matrix, with little effect on chemotactic migration.

### Myoferlin Depletion Alters MMP Expression

**Figure 6:**
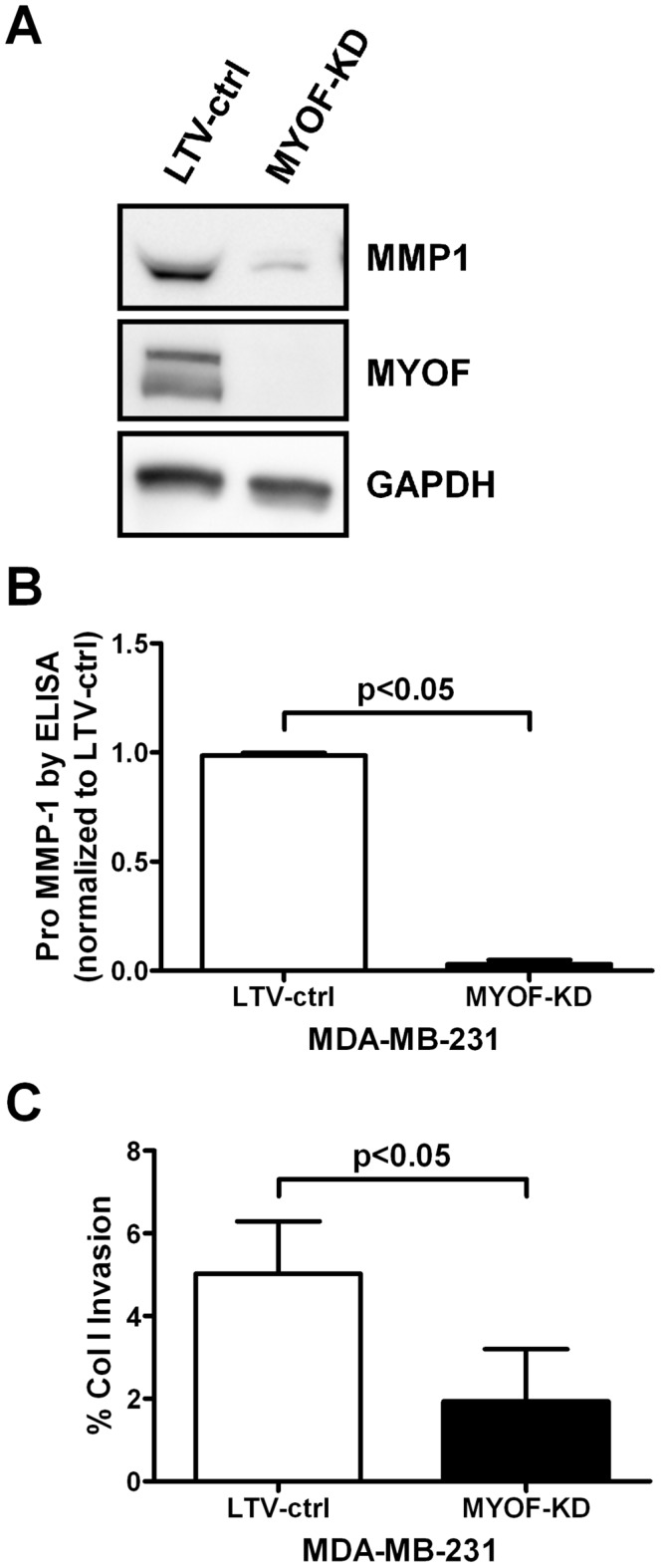
Myoferlin depletion attenuates MMP1 expression and collagen I invasion capacity of MDA-MB-231 cells. (A) Representative (*n* = 3) immunoblotting results of secreted MMP1 in 24 h serum starved supernatant of MDA-MB-231 lentiviral control (LTV-ctrl) and myoferlin knockdown (MYOF-KD) cells. Recombinant human matrix metalloproteinase-1 was used as a standard. Verification of myoferlin knockdown was done in the corresponding cell lysates with GAPDH as a loading control. (B) Secreted pro-MMP1 was evaluated in 231^LTV-ctrl^ and 231^MYOF-KD^ cells (*n* = 2) using ELISA (mean ± s.d., Kruskal-Wallis test/Dunn's multiple comparison analysis). (C) Results from Boyden chamber invasion assays using a coating of 3 mg/ml of rat tail collagen I to evaluate the invasive capacity of 231^LTV-ctrl^ and 231^MYOF-KD^ cells (*n* = 3, mean ± s.d., Kruskal-Wallis test/Dunn's multiple comparison analysis).

The reduced invasive capacity of 231^MYOF-KD^ cells led us to study whether proteins associated with tumor-microenvironment interactions may be altered following MYOF depletion. Specifically, given that MYOF has been implicated in exocytosis [37], we hypothesized that its depletion might decrease the secretion of matrix metalloproteinases (MMPs) and/or tissue inhibitors of MMPs (TIMPs), as these are vital endopeptidases for degrading ECM constituents [38]. To that end, we collected 24 hour conditioned, serum-free supernatants from 231^LTV-ctrl^ and 231^MYOF-KD^ cells and performed an array-based multiplex sandwich ELISA to screen the expression of several of these endopeptidases and their regulators (Figure S5). We observed a reduction in the secretion of MMP-1, -2, -3, and -8 from 231^MYOF-KD^ cells (MMP-2, -3, and -8 were undetectable in 231^MYOF-KD^ cells). MMP-13 levels were not drastically affected, while in contrast, levels of MMP-9 and -10 were elevated in 231^MYOF-KD^ cells. In addition, the 231^MYOF-KD^ cells had reduced levels of TIMP-4, but no notable changes in TIMP-1 and -2 releases were evident. Of the detectable MMPs, the most pronounced change was the decrease in secreted MMP1. However, given the small sample size of this screening experiment (*n* = 1), statistical analysis could not be performed to validate the significance of the observed fold changes. Therefore, to validate the change in MMP1, multiple biological replicates of concentrated 24 hour supernatant from 231^LTV-ctrl^ and 231^MYOF-KD^ cells were subjected to immunoblotting (Figure 6A), and secreted MMP1 was verified to be decreased in 231^MYOF-KD^ cells compared to controls. We also confirmed the diminution (∼97%) of pro-MMP1 secretion into the extracellular milieu by solution-phase ELISA of 231^LTV-ctrl^ and 231^MYOF-KD^ cells (Figure 6B). To confirm the functional association between MYOF knockdown and MMP1 depletion, we investigated the invasive capacity of 231^MYOF-KD^ cells through a collagen I matrix, since fibrillar collagen is a key substrate for the MMP1 enzyme [39] and Matrigel lacks collagen I [40]. In support of these previous observations, 231^MYOF-KD^ cells exhibited a ∼62% reduction in invasion through collagen I matrix compared with 231^LTV-ctrl^ cells (Figure 6C).

We next tested whether MMP1 expression was diminished at the transcriptional level using qRT-PCR. Surprisingly, MMP1 mRNA levels were reduced over an average 18.8-fold in 231^MYOF-KD^ cells when compared with the 231^LTV-ctrl^ cells (Figure S6). This finding of MMP1 mRNA down-regulation in 231^MYOF-KD^ cells was striking, as there have been no reports from other laboratories suggesting that MYOF may contribute to gene expression regulation. Since the diminution of MYOF in the MDA-MB-231 cells was mediated by shRNA (which mimics the function of microRNA molecules [41]), we recognized the potential that MMP1 mRNA could be down-regulated as an off-target effect of the RNAi targeting construct. We therefore screened additional MYOF-targeting shRNA constructs. Although two constructs (Figure S3) yielding partial MYOF knockdown (mean 64–66% knockdown compared with controls, *n* = 4) were not associated with reductions in MMP1 (data not shown), an additional construct (#320398) that targets the coding sequence of MYOF (Figure S3) resulted in a mean of 91% MYOF knockdown (*n* = 3) and was also associated with significant reductions in MMP1 release (Figure S7A and B).

In addition, we performed an extensive *in silico* analysis to examine potential off-target effects of construct #10628. A search through the human nucleotide database for sequences similarity to the 21 nucleotide sequence of #10628 did not reveal MMP1 as a transcript with significant alignment (BLASTN version 2.2.24+) [42]. A search through the microRNA database TargetScan Custom (http://www.targetscan.org/, release 5.1, accessed October 10, 2010) using the 2–8 mer sequence of “CTCTACT” from the #10628 construct did not show MMP1 to be a potential target. Construct #10628 is from a clone cataloged by the RNAi Consortium (http://www.broadinstitute.org), and a search through the consortium database did not show the MYOF shRNA construct as a non-targeting clone with matching transcripts in the MMP1 gene. Finally, the construct sequence was placed into the miRBase [43] to search against known miRNAs. There were no matches of the sequence against mature miRNAs, and all matches to stem-loop sequences using the SSEARCH method were from non-human species (silk worm, mosquito, and platypus) and were not significant (E-value >>0.01).

We did additional PCR screening using a commercial extracellular matrix and adhesion molecules array to profile the expression of MMP genes (Table S2). The screen validated the MMP1 PCR results showing down-regulation of MMP1 mRNA in 231^MYOF-KD^ cells. Specifically, of the four MMPs (MMP1, 10, 11, 14) expressed at reasonable levels in the MDA-MB-231 cells (i.e. Cp value <30), only MMP1 was found to be significantly regulated (≥ ±2 fold-change) at the transcription level by MYOF-depletion.

## Discussion

We provide *in silico* and experimental evidence that MYOF plays a critical and previously unrecognized role in breast tumor cell invasion. Mining of transcriptome and proteome databases from breast cancer cell lines revealed higher levels of MYOF mRNA and protein in the more invasive lines compared with poorly invasive breast tumor cells or normal mammary epithelial cells (i.e., MCF-10A). Published microarray datasets using breast cancer cell lines [34] also support greater MYOF expression in the invasive basal B group compared with the luminal subtype. In a separate study, MYOF was found to be 1 of 39 genes selectively over-expressed in breast carcinoma using 20 human breast cancer cases [28]. These data suggested to us that MYOF is a potentially important protein in breast cancer biology.

The premise that MYOF contributes to cancer invasion was based on several reports implicating a role for ferlin proteins in intracellular vesicle trafficking, including cell motility. For example, Ward and colleagues demonstrated that temperature-sensitive mutations in the ancestral *fer-1* gene in *C. elegans* led to fertility impairments yielding immotile spermatozoa secondary to defects in vesicle recycling at the leading edge of the cell [15], [44]. Mechanistically, mutations in C2 domains of *fer-1* altered calcium sensitivity and subsequently impeded calcium-dependent fusion of intracellular vesicles to the plasma membrane that drives the amoeboid movement of the roundworm sperm cells [15]. Vesicle trafficking has been implicated in several important steps during cell motility [9]. Based on these observations, coupled with the observation that MYOF expression was increased in breast cancer specimens and breast cancer cell lines with high invasive potential, we hypothesized that MYOF depletion would impair the motility of breast cancer cells. And this appeared to be the case. End-point Boyden chamber assays accessing cell motility initially detected no overall change in migration during a 24 hour assay period. This was somewhat surprising in light of the work of Achanzar *et* *al.* [45] demonstrating that mutation of *fer-1* resulted in profound defects in amoeboid migration of *C. elegans* sperm cells. However, recent work from our lab using live-cell imaging clearly shows that MYOF depletion leads to fundamental changes in the mode of breast tumor cell motility (Volakis *et* *al.*, in preparation, *J Biomechanics*), suggesting that MYOF loss also contributes to disturbances in cell migration on two-dimensional surfaces (work in progress).

Tumor cell invasion into distant tissues to establish secondary malignancies is a hallmark of cancer metastasis and cancer-related death [4]. In many cases, enhanced tumor cell migration as well as local and distant invasion are preceded by an EMT that converts sessile epithelial cells into actively migratory and invasive cells [46], [47]. In order for efficient migration and invasion to occur, in many instances cancer cells must secrete several MMPs for basement membrane and stromal ECM degradation [48]. We probed the invasive capacity of breast tumor cells (MDA-MB-231) following shRNA directed depletion of MYOF (shRNA^MYOF^), and noted a profound deficit in the ability of cells to invade through Matrigel (basement membrane mimic) [49] and collagen type I (stromal ECM mimic) [50]. These observations indicated that MYOF disruption may perturb one or more MMPs that are essential for effective cell invasion through the ECM or collagen I [48]. Indeed, screens of several MMPs using qRT-PCR and protein immunoassay arrays suggested that MMPs-1, 3, 8, 12, 13, 14, and 16 were down-regulated in MDA-MB-231 cells in which MYOF was knocked down by RNAi (Figure S5 and Table S2). Interestingly, MMP-9 appeared to be up-regulated under these same conditions, indicating the MYOF depletion results in selective changes in MMPs.

To evaluate the effect of abolishing MYOF on MMP expression, we probed MMP-1 further, because our data revealed a nearly complete loss of MMP-1 when shRNA^MYOF^ was expressed in MDA-MB-231 cells. Quantitative RT-PCR and Western blotting showed essentially no detectable MMP-1 mRNA or protein in 231^MYOF-KD^ cells. Moreover, we detected virtually no pro-MMP-1 reactive protein by ELISA. Thus, it appears that MYOF may play a role in MMP gene expression in addition to other mechanisms it may influence, such as MMP secretion via vesicle trafficking. We are currently exploring the mechanistic basis for this apparent alteration in MMP gene expression in human breast tumor cells.

To authenticate that our shRNA results were not due to off-target effects on the MMP mRNAs, we conducted an extensive *in silico* analysis using several software packages available in the public domain and verified that none of the shRNA^MYOF^ constructs predicted the targeting of any nucleic acid sequences within the human MMP-1 gene. In addition, we found MMP1 expression to be also down-regulated when MYOF expression was knocked down using an alternative shRNA construct with similar knock down efficiency.

Another unanticipated finding in the current studies was the partial reversion of the mesenchymal-like shape in wild-type and control MDA-MB-231 cells to a more epithelial-like phenotype in 231^MYOF-KD^ cells. We verified this potential MET event using immunofluorescence, scanning, and atomic force microscopy. In addition, we also demonstrated the likelihood of an MET in the setting of depleted MYOF using immunoblotting that revealed down-regulation of fibronectin and vimentin and up-regulation of E-cadherin.

In a recent paper from our group, MYOF depletion was shown to lead to the down-regulation of the phosphorylation of several receptor tyrosine kinases, including EphB4, FGFR2, Hck, IGF-IR, JAK2, TXK, VEGFR2 [30]. This observation is in agreement with the work of Demonbreun *et* *al.* demonstrating that loss of MYOF led to impaired insulin-like growth factor I receptor (IGF-IR) signaling in a murine model [51]. In separate reports, MYOF was shown to be required for Tie-2 (angiopoietin-1 receptor) activation [25] and vascular endothelial growth factor receptor 2 (VEGFR-2) expression in vascular endothelial cells [24]. Moreover, Sharma *et* *al.*, showed that disruption of dysferlin, a close molecular cousin of MYOF, in endothelial cells led to polyubiquitination and proteasomal degradation of platelet endothelial cellular adhesion molecule-1 (PECAM-1/CD31) signaling [52].

These data, when considered collectively and in the context of our breast tumor cell investigations, implicate MYOF as a critical participant in key processes within the intracellular vesicle trafficking mechanism including cell motility, MMP expression/secretion, and RTK activation. Our work is the first examination of the role of MYOF in cancer cell biology and may shed light on essential features of vesicle shuttling of important cargos within tumor cells that participate in cancer progression and metastasis.

## Materials and Methods

### Cell Culture Methods and Cell Line Authentication

Human MCF-10A (generously provided by Dr. Joan Brugge, Boston, MA, [32], [53]), MCF-7 (HTB-22, ATCC, Bethesda, MD), MDA-MB-231 (HTB-26, ATCC), T47D (HTB-133, ATCC), and BT549 (generous gift from Dr. Lisa Yee, Columbus, OH; HTB-122, ATCC) cells were used in this study. MCF-10A cells were maintained in Dulbecco's Modified Eagle Medium Ham’s F12 (DMEM/F12, Gibco, Carlsbad, CA) supplemented with 5% horse serum (Gibco), 20 ng/ml hEGF (PeproTech, Rocky Hill, NJ), 0.5 µg/ml hydrocortisone (Sigma-Aldrich, St. Louis, MO), 100 ng/ml cholera toxin (Sigma-Aldrich), 10 µg/ml bovine insulin (Sigma-Aldrich), and 1% penicillin/streptomycin (Gibco). MCF-7 and MDA-MB-231 cells were maintained in DMEM with 4.5 g/L D-glucose supplemented with 10% fetal bovine serum (FBS, Gibco). T47D and BT549 cells were cultured in RPMI-1640 with 10% FBS.

The cell lines used in this study were authenticated by DNA profiling using short tandem repeat (STR) analysis on a PowerPlex 1.2 System (Promega, Madison, WI) at John Hopkins University (Fragment Analysis Facility, Baltimore, MD). Furthermore, to rule out cell line contamination as the cause for the morphology changes seen in MDA-MB-231 myoferlin-deficient cells (231^MYOF-KD^), the identity of the 231^MYOF-KD^ cells as MDA-MB-231s was also authenticated through short tandem repeat profiling.

### Lentiviral shRNA Transduction

Recombinant lentiviral particles containing non-target control shRNA and human myoferlin targeted shRNA (TRCN0000010628, TRCN0000010630, TRCN0000001522, and TRCN0000320398, Figure S3) in the pLKO.1 vector were purchased from Sigma-Aldrich (MISSION®). The myoferlin constructs are referred to as #10628, #10630, #1522 and #320398, respectively, in the body of the manuscript. For lentiviral transduction, cells were seeded in 24-well culture plates and incubated overnight at 37°C in 5% CO_2_ in a humidified atmosphere. Media was replaced with media containing 8 µg/ml hexadimethrine bromide (Sigma-Aldrich) and lentiviral particles were added to subconfluent at a multiplicity of infection (MOI) of at least 1. After overnight incubation (37°C, 5% CO_2_), virus-containing supernatant was replaced with complete media and incubated overnight. Transduced cells were selected in media containing an appropriate puromycin concentration as predetermined by a puromycin kill curve.

### RNA Extraction and Quantitative Real Time-Polymerase Chain Reaction (qRT-PCR)

Total RNA was extracted using TRIzol® reagent (Invitrogen, Carlsbad, CA) according to the manufacturer’s protocol up to the chloroform extraction and centrifugation step. The resulting aqueous phase was mixed with an equal volume of 70% ethanol and applied to an RNeasy mini column (QIAGEN, Valencia, CA) and processed according to the manufacturer's protocol with on column DNAse digestion. Total RNA was quantified by UV absorbance at 260 nm and 280 nm on a NanoDrop 2000 (Thermo Scientific, Waltham, MA), and 1–2 µg total RNA from each sample were reverse-transcribed to cDNA using oligo-dT18 and random hexamer primers with a Transcriptor First Strand cDNA Synthesis Kit (Roche Applied Science, Indianapolis, IN). Quantitative RT-PCR was performed using an equal amount of cDNA per sample on a LightCycler® 480 II System (Roche Applied Science) using primer-probe sets specific to MYOF, MMP1, RLPO and 18 S rRNA (Applied Biosystems, Carlsbad, CA) with the LightCycler® 480 Probes Master Mix, and results analyzed with the LightCycler® 480 Software.

### Immunofluorescence

MDA-MB-231 cells cultured on glass coverslips and serum-starved for 24 h were fixed for 1 h in 4% (w/v) paraformaldehyde/PBS, permeabilized for 15 min with 0.2% (v/v) Triton X–100/PBS, and blocked for 1 h in 5% normal goat serum/1% nonfat dry milk/PBS. Samples were incubated with antibodies directed against myoferlin (1∶400, Sigma) overnight at 4°C. After washing, cells were exposed to fluorochrome-conjugated secondary antibodies (Molecular Probes). After further stringent washing in PBS, actin cytoskeleton was stained with AlexaFluor 488-phalloidin (Molecular Probes) for 20 min. Nuclei were stained and cells mounted with ProLong® Gold Antifade Reagent with DAPI (Invitrogen) and visualized with confocal laser scanning microscopy (Olympus FV 1000 Spectral Confocal system; Olympus America Inc., Center Valley, PA).

### Atomic Force (AFM) and Scanning Electron Microscopy (SEM)

MDA-MB-231 lentiviral control and myoferlin-deficient cells were seeded in 60 mm cell culture dishes (5–8×10^5^ cells), and allowed to grow to 80–90% confluence for AFM analysis. Prior to AFM analysis, culture media was exchanged for CO_2_ independent media (Invitrogen) supplemented with 1% FBS, 1% Penicillin/Streptomycin and 4 mM L-Glutamine, for optimal cell survival during the atomic force measurements. A Bioscope II instrument (Veeco, Plainview, NY) mounted on the stage of an Axiovert 200 inverted optical microscope (Zeiss, Oberkochen, Germany) was used in contact mode for live cell imaging. Silicon nitride (SiN) triangular cantilevers (Veeco) 200 µM in length with a tip angel θ = 35° and a nominal spring constant k of 0.01 N/m were used to image the cells. Image acquisition was done at a scanning rate of 0.5 Hz with 512×512 lines resolution for recording the height and the deflection channels. All images were recorded with a scan size area of 70–80 µm^2^ covering ∼5 cells, with 4–5 areas completed per cell type. The experiments were repeated three times and the data were analyzed using version 7.30 of the NanoScope software (Veeco).

For SEM, cells cultured on thermoplastic coverslips (Thermanox™, Nunc, Waltham, MA) were fixed with 3% glutaraldehyde, post-fixed with 1% OsO_4_ and dehydrated through an ethanol and hexamethyldisilazane series. Samples were mounted onto SEM studs and prepared for SEM using a Pelco Model 3 sputter coater with gold-palladium (0.07 mbar, 17 mA, 110 s) and imaged with an FEI Nova NanoSEM microscope (FEI Company, Hillsboro, OR).

### Protein Extraction and Immunoblotting

Cells were rinsed with PBS and lysed for 30 min in cold RIPA lysis buffer supplemented with protease inhibitor cocktail (Sigma-Aldrich) and 1 µg/ml of pepstatin A (USB, Santa Clara, CA). Cell lysates were centrifuged for 10 min at 17,000×*g* and 4°C. Supernatant aliquots were assayed for protein concentration using the BCA Protein Assay (Pierce, Rockford, IL) with bovine serum albumin as a standard. Total protein from each lysate (30 µg per lane) were resolved by SDS-PAGE and transferred to nitrocellulose membranes. Non-specific binding was blocked by incubation in Tris-buffered saline (pH 8) containing 0.1% Tween-20 and 5% non-fat dry milk. Membranes were probed with primary antibodies against: β-actin (Santa Cruz Biotechnology, Santa Cruz, CA), GAPDH (Chemicon, Billerica, MA), myoferlin (Sigma-Aldrich), E-cadherin (EP700Y, Abcam, Cambridge, MA), vimentin (Sigma-Aldrich V6630), or MMP1 (R&D Systems, Minneapolis, MN) diluted in blocking solution. After washing, membranes were exposed to horseradish peroxidase-conjugated secondary antibodies and immune complexes were revealed with SuperSignal West Femto chemiluminescent substrate (Pierce), visualized using the VersaDoc Imaging System and analyzed with Quantity One analysis software (Bio-Rad, Hercules, CA).

### Supernatant Collection and Immunoblotting

Cell conditioned media were collected after the cells were plated at equal densities in 100 mm dishes, then serum starved in 4 ml of basal media for 24 h. The conditioned media were concentrated 50-fold with Amicon Ultra-4 10-kDa centrifugal filters (Millipore, Billerica, MA), and equal volumes of concentrated media were reconstituted in sample buffer and resolved by SDS-PAGE. Human recombinant MMP1 (rhMMP-1) from conditioned media of rhMMP-1 expressing NS0 mouse myeloma cells (WBC024; R&D Systems) was used as a positive control. Gels were processed per immunoblotting procedures as detailed in the previous section, using anti-human mouse monoclonal antibodies against MMP-1 (MAB901; R&D Systems) and fibronectin (610077; BD Transduction Laboratories, San Jose, CA).

### MMP Expression Array and MMP1 ELISA

ECM proteins (MMP1, -2, -3, -8, -9, -10, and -13 and TIMP1, -2, -3, and -4) in conditioned, serum starved supernatants of lentiviral-transduction control (MDA-MB-231^LTV-ctrl^) and MDA-MB-231^MYOF-KD^ cells were determined by an antibody array (RayBio® human matrix metalloproteinase antibody array 1, RayBiotech, Inc., Norcross, GA) following the manufacturer's protocol. Each array membranes contained duplicates for each ECM protein along with positive and negative controls. The blots were imaged and quantified by densitometry (Quantity One software, Bio-Rad), and values normalized to total protein of the corresponding cell lysates.

Secreted pro-MMP-1 in cultured media of cells was detected by quantitative ELISA (DMP100; R&D Systems). Conditioned cultured media were collected by plating equal densities of each cell type in 100 mm culture dishes followed by serum-starvation for 24 h. Unconcentrated, conditioned media samples were run in duplicate in the ELISA and processed according to manufacturer’s instructions. Colorimetric results were read at 450 nm with a wavelength correction of 570 nm in a MRX Microplate Reader (Dynex Technologies, Chantilly, VA). MMP-1 concentrations were determined by interpolation using human recombinant pro-MMP-1 as a standard. In cases where the secreted pro-MMP1 was undetectable, it was assigned the assay’s lowest limit of detection (0.021 ng/ml).

### MTS Proliferation Assay and Cell Cycle Analysis

Cells were plated in 96-well plates at a density of 2500 and/or 5000 cells per well and assayed at 24, 48, and 72 h after plating using the CellTiter 96 AQueous Non-Radioactive Cell Proliferation Assay (Promega), according to the manufacturer's instructions.

For flow cytometric analysis of MYOF depleted breast cancer cells, cells were fixed in 70% ethanol and stained in PBS containing 0.1% Triton X-100, 200 µg/mL of RNase, and 50 µg/mL propidium iodide (Sigma-Aldrich). DNA content was measured on a FACS Calibur or LSR II flow cytometer (Becton-Dickinson)), and data were acquired and analyzed using flow cytometer software (BD). Each analyzed sample contained at least 1×10^6^ cells.

### Migration and Invasion Assays

Cells were washed with PBS, trypsinized, pelleted and resuspended in serum-free DMEM. Cells were then seeded onto Boyden Chamber inserts (8 µm pores; Millipore) at a density of 7.5×10^4^ cells in 24-well plates with 10% FBS containing DMEM in the bottom chamber as a chemo-attractant. In parallel, input controls were seeded with the same number of cells in the same volumes of serum-free and 10% FBS media without inserts. Cells were incubated (37°C, 5% CO_2_) for 24 hours. The inserts were processed by rinsing in PBS and fixation with 3.7% formaldehyde containing 0.05% crystal violet for 10 min. After repeated washes with PBS and distilled water the chambers were air-dried. Migrated cells on the bottom of the inserts were collected with cotton swabs and placed into Eppendorf tubes. Crystal violet dye was extracted from the Q-tips and input control wells with 80% methanol for 30 min, and quantified at 570 nm. Percentage migration was calculated using the ratio of the migrated cells over the total cells (input control) to determine the percentage of cell migration. For invasion, the Boyden Chamber assay was conducted in the same manner but with the addition of 20 µl of growth factor reduced Matrigel (BD) or 3 mg/ml collagen I (rat tail, Gibco) to the top of the insert and allowed to gel for 1 h at 37°C, 5% CO_2_.

### Quantitative Reverse Transcription PCR Array

RT2 Profiler™ PCR array (SABiosciences, Frederick, MD) was performed for EMT-associated genes (cat #PAHS-090) and human extracellular matrix and adhesion molecules (cat # PAHS-013) following manufacturer's protocol. Briefly, total RNA was extracted as described in the qRT-PCR section from lentiviral control and myoferlin-deficient MDA-MB-231 cells that had been in culture for 10 consecutive passages. Complementary DNA was generated from 1 µg of total RNA per cell type using random hexamers and oligo-dT18 primers as part of the RT2 First Strand Kit (SABiosciences). Equal amounts of dilute cDNA was mixed with LightCycler® 480 SYBR Green I Master mix (Roche) and aliquoted to each well of the PCR array plate containing pre-filled gene-specific primer sets, and PCR was performed according to manufacturer’s instructions for the Roche LightCycler® 480. The LightCycler® 480 Software (Roche Applied Science) was used to calculate the threshold cycle (crossing point, Cp) values for all the transcripts in the array. The Cp values were exported into a spreadsheet-based PCR array data analysis template (SABiosciences) to calculate fold changes in gene expression using the ΔΔCt method.

### Statistical Analysis

Graphical representation and statistical analysis of data were done using Prism version 5 (GraphPad). Error bars represent standard deviation in all cases, unless differently noted. Statistical significance was determined by appropriate statistical tests following normality testing of the data, using the KS, D'Agostino & Pearson omnibus and/or Shapiro-Wilk normality tests. Comparisons between 2 groups with Gaussian distribution were carried out with the 2-tailed student t-test for unpaired samples, while those among 3 or more groups were carried out with one-way ANOVA followed by Tukey's Multiple Comparison Test. Data exhibiting a non-Gaussian distribution were analyzed with either the Mann Whitney statistical test (2 groups) or the Kruskal-Wallis with Dunn’s multiple comparison post-test (3 or more groups). For analysis, results were regarded as significant if *p* values were less than 0.05.

## Supporting Information

Figure S1 **Analysis of the myoferlin gene expression in breast cancer cells.** Graphical representation (mean ± s.d.) of expression data from two microarray probe sets for myoferlin reported in the study by Neve and colleagues [34], showing a higher expression (Kruskal-Wallis test/Dunn's multiple comparison analysis) in the Basal B cells compared with the luminal cells.
(TIF)

Figure S2 **Graphical representation from ArrayExpress gene expression atlas (accession # E-TABM-276) of MYOF mRNA expression level in breast tissue samples from healthy patients and patients with invasive ductal carcinoma.**
(TIF)

Figure S3 **Myoferlin lentiviral constructs.** Target and sequence information of lentiviral constructs used to generate myoferlin-deficient cell lines. Bold letters indicate coding sequence.
(TIF)

Figure S4 **Proliferation not affected by myoferlin depletion in MDA-MB-231 cells.** Growth curves (A) and cell cycle analysis (B) of wild type (WT), lentiviral control (LTV-ctrl) and myoferlin knockdown (MYOF-KD) MDA-MB-231 cells. Statistical analysis on the proliferation curves (*n* ≥4, mean ± s.d.) were done using linear regression on the log transformation of the OD readings (*p* = 0.43), showing an insignificance difference among the proliferation rate of the cells. The cell cycle analysis (*n* ≥4, mean ± s.d.) also showed an insignificant difference in cell proliferation (Gaussian approximation P value of 0.96, Kruskal-Wallis/Dunn's multiple comparison post-test).
(TIF)

Figure S5 **Myoferlin depletion in MDA-MB-231 cells alters the secretion of matrix metalloproteinases (MMPs) and tissue inhibitors of MMPs (TIMPs).** Antibody membrane-based array detecting various MMPs and TIMPs was used to screen whether myoferlin depletion changes the secretion of MMPs and TIMPs. 231^LTV-ctrl^ and 231^MYOF-KD^ cells were serum starved for 24 h, and culture supernatants collected and analyzed for extracellular matrix proteins. The intensity of the quantified signals was normalized to 231^LTV-ctrl^ cells, and results are expressed as fold changes.
(TIF)

Figure S6 **MMP1 mRNA expression attenuated by myoferlin depletion in MDA-MB-231 cells.** Relative levels of MMP1 mRNA in lentiviral-control (LTV-ctrl) and myoferlin depleted (MYOF-KD) MDA-MB-231 cells. Levels of 18 S expression were used to normalize the samples. Graph represents fold change normalized to MMP1 levels in 231^LTV-ctrl^ cells (*n* = 3, mean ± s.d.), showing a significant depletion of MMP1 mRNA in 231^MYOF-KD^ cells (unpaired t-test, *p* = 0.003).
(TIF)

Figure S7 **Myoferlin depletion in MDA-MB-231 cells by separate shRNA constructs attenuates MMP1 production.** Immunoblotting (A) and ELISA (B) evaluation of secreted MMP1 in 24 h serum starved supernatant of myoferlin depleted MDA-MB-231 cells (constructs #10628 and #320398). Three replicate samples of construct #320398 were ran (A–C). ELISA results (*n* = 3, mean ± s.d.) show significant depletion of secreted MMP1 in both myoferlin depleted MDA-MB-231 cells (one way ANOVA/Tukey's Multiple Comparison Test, *p*<0.01) when compared with the lentiviral control cells.
(TIF)

Table S1 **Reported is an alphabetical listing of genes with Cp values less than 30 cycles for at least one of the samples and with fold changes of** ***≥ ±*** **2-fold.** Fold change for genes up-regulated in MYOF-deficient MDA-MB-231 cells are in **bold**, while the down-regulated genes are in *italics*.
(DOCX)

Table S2 **Reported are fold changes at the mRNA level, along with each MMP’s known ECM substrates** [**54**] **.** MMPs with Cp values less than 30 cycles for at least one of the samples are formatted in **bold** (MMP1, MMP10, MMP11, and MMP14). Fold change for genes down-regulated in MYOF-deficient MDA-MB-231 cells are in *italics*.
(DOCX)
