# Supplementary figures and images for: Myoferlin Depletion in Breast Cancer Cells Promotes Mesenchymal to Epithelial Shape Change and Stalls Invasion
Source: PLoS One. 2012 Jun 27;7(6):e39766. doi: 10.1371/journal.pone.0039766 (PMC3384637; doi:10.1371/journal.pone.0039766)

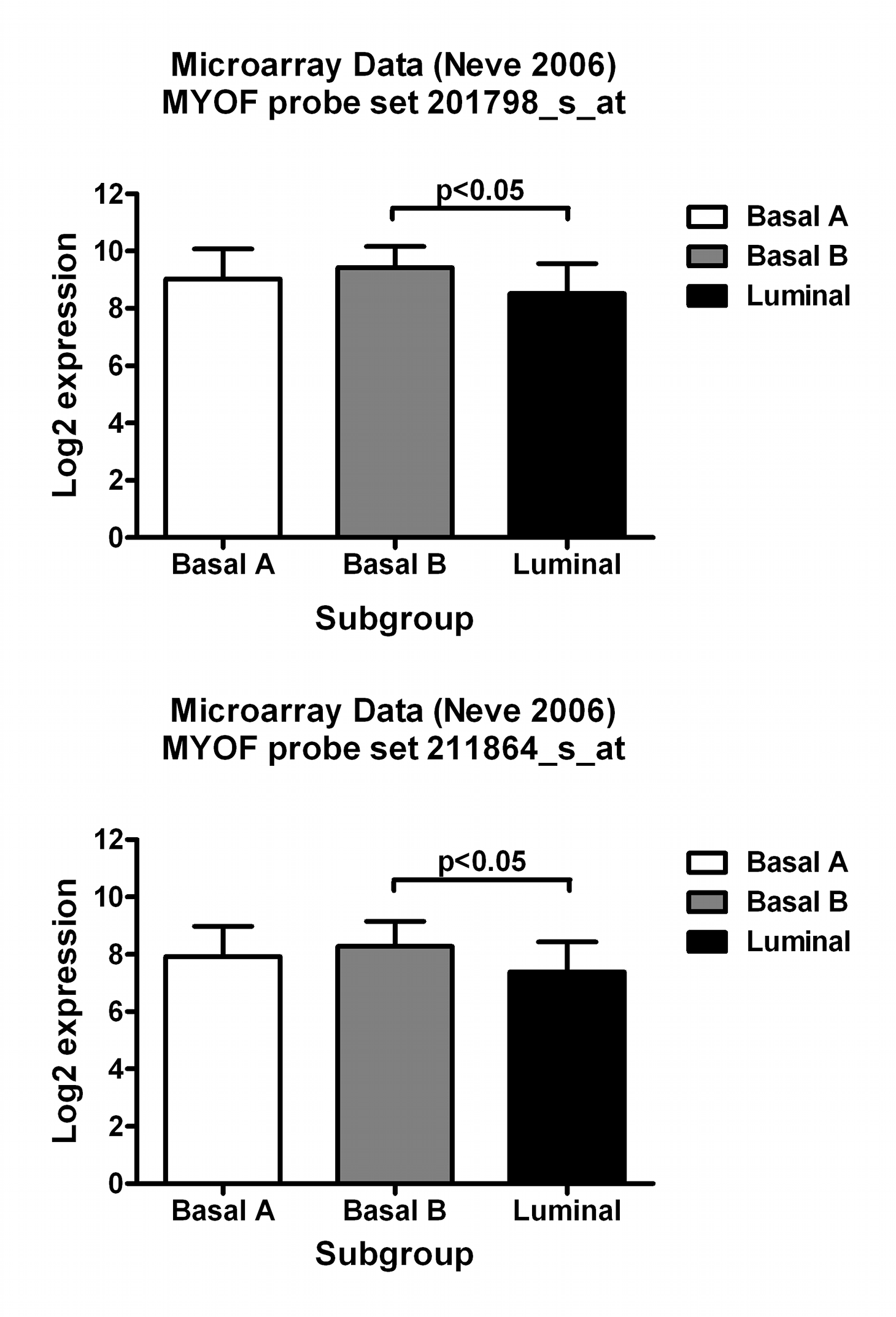

Supplement: Figure S1 — Analysis of the myoferlin gene expression in breast cancer cells. Graphical representation (mean ± s.d.) of expression data from two microarray probe sets for myoferlin reported in the study by Neve and colleagues [34], showing a higher expression (Kruskal-Wallis test/Dunn's multiple comparison analysis) in the Basal B cells compared with the luminal cells. (TIF) [file pone.0039766.s001.tif]

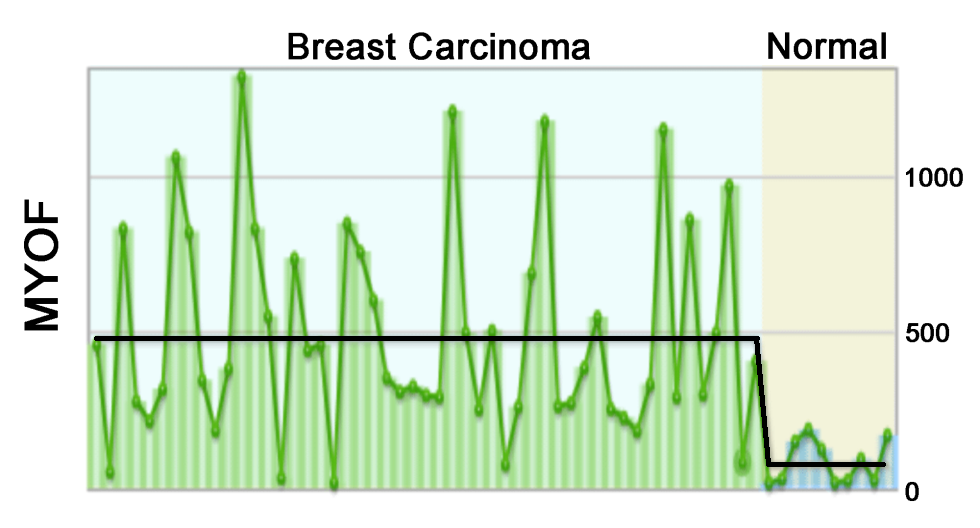

Supplement: Figure S2 — Graphical representation from ArrayExpress gene expression atlas (accession # E-TABM-276) of MYOF mRNA expression level in breast tissue samples from healthy patients and patients with invasive ductal carcinoma. (TIF) [file pone.0039766.s002.tif]

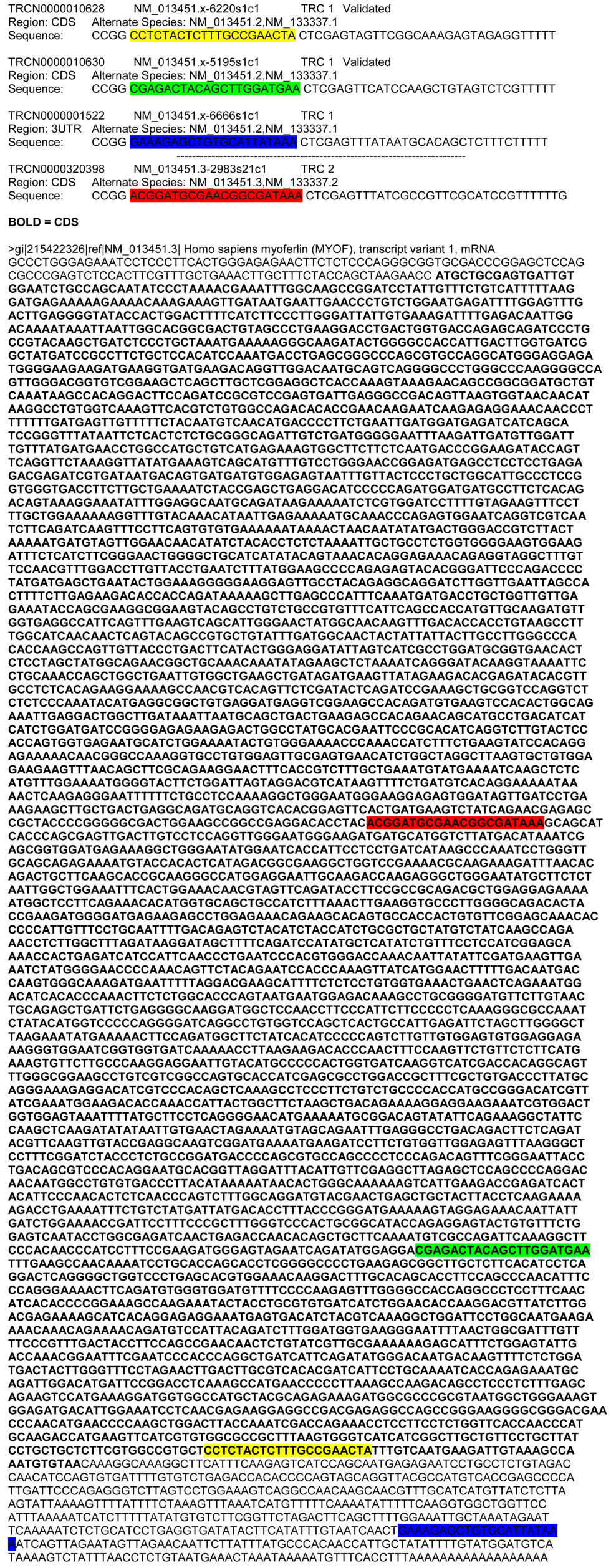

Supplement: Figure S3 — Myoferlin lentiviral constructs. Target and sequence information of lentiviral constructs used to generate myoferlin-deficient cell lines. Bold letters indicate coding sequence. (TIF) [file pone.0039766.s003.tif]

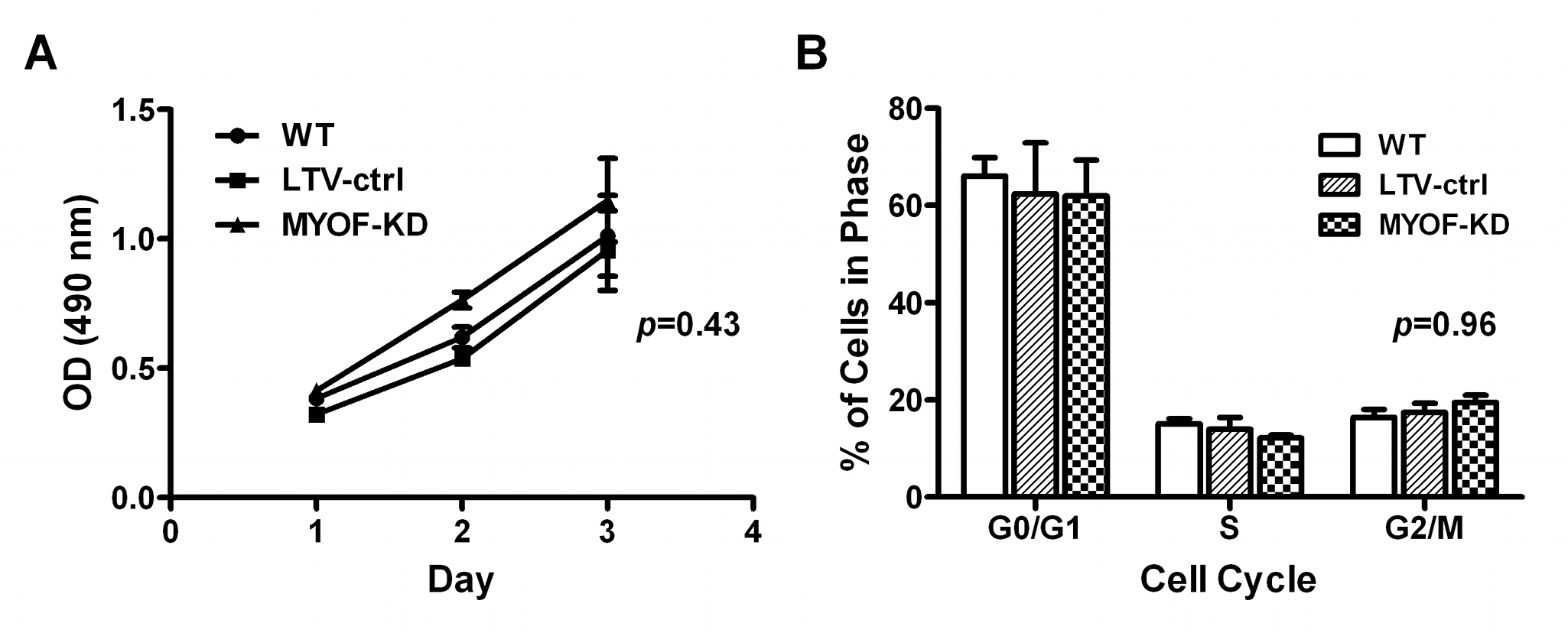

Supplement: Figure S4 — Proliferation not affected by myoferlin depletion in MDA-MB-231 cells. Growth curves (A) and cell cycle analysis (B) of wild type (WT), lentiviral control (LTV-ctrl) and myoferlin knockdown (MYOF-KD) MDA-MB-231 cells. Statistical analysis on the proliferation curves (n ≥4, mean ± s.d.) were done using linear regression on the log transformation of the OD readings (p = 0.43), showing an insignificance difference among the proliferation rate of the cells. The cell cycle analysis (n ≥4, mean ± s.d.) also showed an insignificant difference in cell proliferation (Gaussian approximation P value of 0.96, Kruskal-Wallis/Dunn's multiple comparison post-test). (TIF) [file pone.0039766.s004.tif]

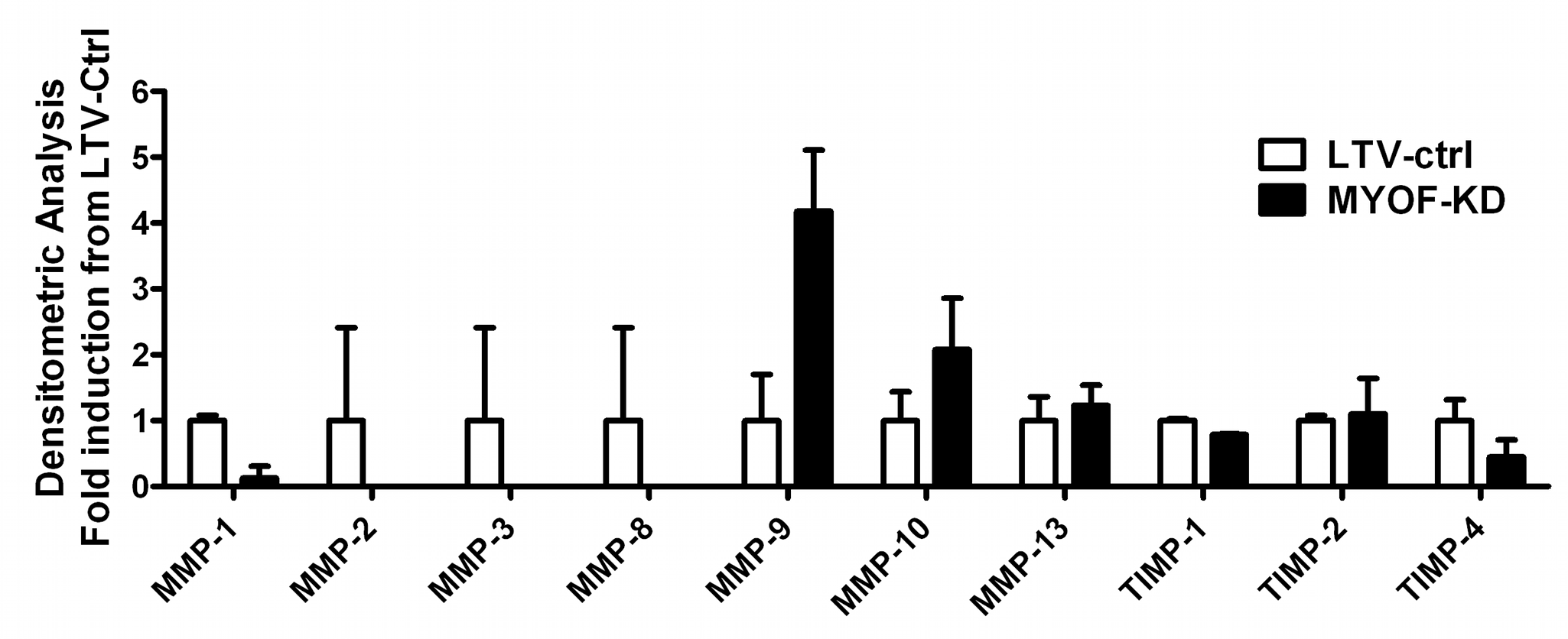

Supplement: Figure S5 — Myoferlin depletion in MDA-MB-231 cells alters the secretion of matrix metalloproteinases (MMPs) and tissue inhibitors of MMPs (TIMPs). Antibody membrane-based array detecting various MMPs and TIMPs was used to screen whether myoferlin depletion changes the secretion of MMPs and TIMPs. 231LTV-ctrl and 231MYOF-KD cells were serum starved for 24 h, and culture supernatants collected and analyzed for extracellular matrix proteins. The intensity of the quantified signals was normalized to 231LTV-ctrl cells, and results are expressed as fold changes. (TIF) [file pone.0039766.s005.tif]

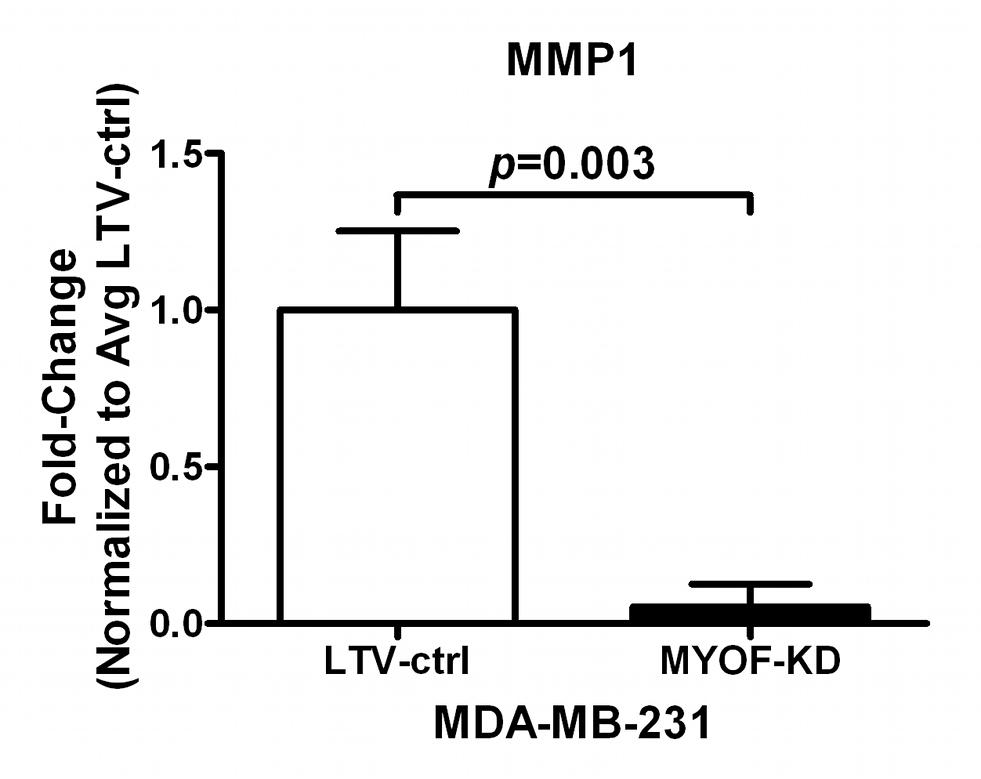

Supplement: Figure S6 — MMP1 mRNA expression attenuated by myoferlin depletion in MDA-MB-231 cells. Relative levels of MMP1 mRNA in lentiviral-control (LTV-ctrl) and myoferlin depleted (MYOF-KD) MDA-MB-231 cells. Levels of 18 S expression were used to normalize the samples. Graph represents fold change normalized to MMP1 levels in 231LTV-ctrl cells (n = 3, mean ± s.d.), showing a significant depletion of MMP1 mRNA in 231MYOF-KD cells (unpaired t-test, p = 0.003). (TIF) [file pone.0039766.s006.tif]

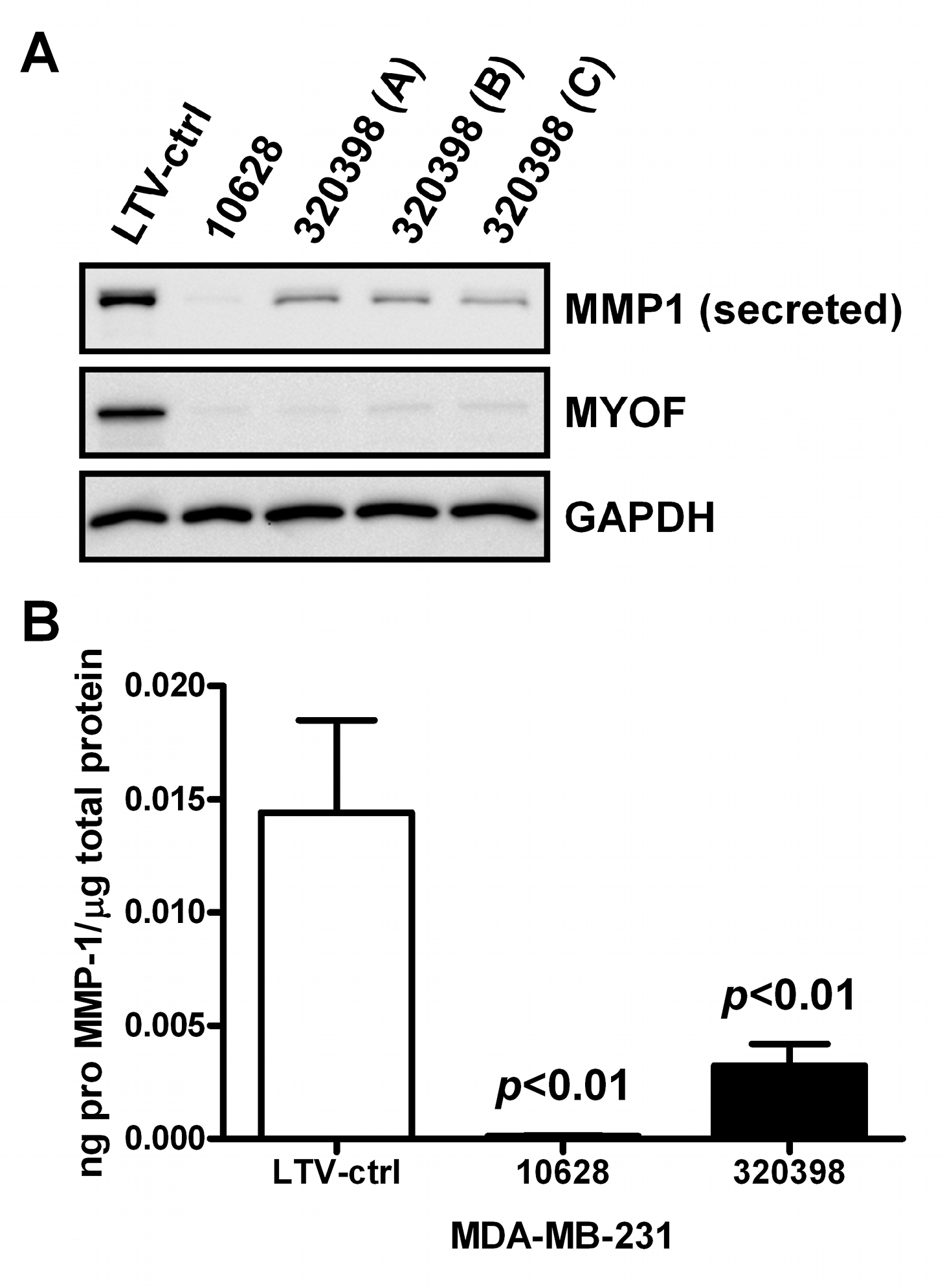

Supplement: Figure S7 — Myoferlin depletion in MDA-MB-231 cells by separate shRNA constructs attenuates MMP1 production. Immunoblotting (A) and ELISA (B) evaluation of secreted MMP1 in 24 h serum starved supernatant of myoferlin depleted MDA-MB-231 cells (constructs #10628 and #320398). Three replicate samples of construct #320398 were ran (A–C). ELISA results (n = 3, mean ± s.d.) show significant depletion of secreted MMP1 in both myoferlin depleted MDA-MB-231 cells (one way ANOVA/Tukey's Multiple Comparison Test, p<0.01) when compared with the lentiviral control cells. (TIF) [file pone.0039766.s007.tif]
