# Supplementary material for: Myoferlin Depletion in Breast Cancer Cells Promotes Mesenchymal to Epithelial Shape Change and Stalls Invasion
Source: PLoS One. 2012 Jun 27;7(6):e39766. doi: 10.1371/journal.pone.0039766 (PMC3384637; doi:10.1371/journal.pone.0039766)
Supplement: Table S1 — Reported is an alphabetical listing of genes with Cp values less than 30 cycles for at least one of the samples and with fold changes of ≥ ± 2-fold. Fold change for genes up-regulated in MYOF-deficient MDA-MB-231 cells are in bold, while the down-regulated genes are in italics. (DOCX) [file pone.0039766.s008.docx]

**Table S1.** PCR array screen of EMT-related genes in 231^LTV-ctrl^ and 231^MYOF-KD^ cells.

| **Gene Symbol** | **Description** | **231^LTV-ctrl^ Cp value** | **231^MYOF-KD^ Cp* value** | **MYOF-KD/**  **LTV-ctrl  Fold Change** |
| --- | --- | --- | --- | --- |
| CDH1 | Cadherin 1, type 1, E-cadherin (epithelial) | 29.47 | 28.21 | **2.5847** |
| DSC2 | Desmocollin 2 | 28.57 | 32.99 | *-19.8353* |
| FN1 | Fibronectin 1 | 21.85 | 23.75 | *-3.4581* |
| JAG1 | Jagged 1 (Alagille syndrome) | 25.54 | 27.46 | *-3.5064* |
| PDGFRB | Platelet-derived growth factor receptor, beta polypeptide | 28.68 | 27.73 | **2.0849** |
| SERPINE1 | Serpin peptidase inhibitor, clade E (nexin, plasminogen activator inhibitor type 1), member 1 | 22.14 | 20.74 | **2.8481** |
| SPARC | Secreted protein, acidic, cysteine-rich (osteonectin) | 31.27 | 28.43 | **7.7275** |
| STEAP1 | Six transmembrane epithelial antigen of the prostate 1 | 24.84 | 26.17 | *-2.3295* |
| TMEM132A | Transmembrane protein 132A | 28.17 | 27.06 | **2.3295** |

* Cp = crossing point, Roche software designation for threshold cycle
