# Supplementary material for: Myoferlin Depletion in Breast Cancer Cells Promotes Mesenchymal to Epithelial Shape Change and Stalls Invasion
Source: PLoS One. 2012 Jun 27;7(6):e39766. doi: 10.1371/journal.pone.0039766 (PMC3384637; doi:10.1371/journal.pone.0039766)
Supplement: Table S2 — Reported are fold changes at the mRNA level, along with each MMP’s known ECM substrates [54] . MMPs with Cp values less than 30 cycles for at least one of the samples are formatted in bold (MMP1, MMP10, MMP11, and MMP14). Fold change for genes down-regulated in MYOF-deficient MDA-MB-231 cells are in italics. (DOCX) [file pone.0039766.s009.docx]

**Table S2.** PCR array screen of MMPs in 231^LTV-ctrl^ and 231^MYOF-KD^ cells.

| **MMP** | **231^LTV-ctrl^ Cp value** | **231^MYOF-KD^ Cp* value** | **MYOF-KD/LTV-ctrl  Fold Change** | **ECM Substrate [54]** |
| --- | --- | --- | --- | --- |
| **MMP1** | 25.48 | 32.70 | *-119.05* | Collagen I/II/III/VII/X/XI, gelatin, entactin, aggrecan, ﬁbronectin, laminin, tenascin, vitronectin |
| MMP2 | 32.75 | 32.53 | 1.46 | Collagen I/III/IV/V/VII/X/XI, gelatin, elastin, ﬁbronectin, laminin, aggrecan, tenascin, decorin, vitronectin |
| MMP3 | 32.02 | 32.75 | *-1.33* | Collagen III/IV/V/VII/IX/X/XI, elastin, laminin, ﬁbronectin, gelatin, aggrecan entactin, decorin, tenascin, vitronectin |
| MMP7 | 32.71 | 33.08 | *-1.03* | Collagen I/IV, aggrecan, laminin, ﬁbronectin, gelatin, entactin, decorin, elastin, tenascin, vitronectin |
| MMP8 | 32.47 | 35.00 | *-4.61* | Collagen I/II/III, aggrecan |
| MMP9 | 32.03 | 30.33 | 4.07 | Collagen IV/V/XI/XIV, decorin, gelatin, elastin, laminin, aggrecan, vitronectin |
| **MMP10** | 30.20 | 29.73 | 1.73 | Collagen III/IV/V, aggrecan, elastin, laminin, fibronectin, gelatin |
| **MMP11** | 26.02 | 26.29 | 1.04 | Not determined |
| MMP12 | 33.46 | 34.06 | *-1.21* | Collagen I/IV, aggrecan, decorin, gelatin, elastin, ﬁbronectin, laminin, vitronectin, entactin |
| MMP13 | 31.55 | 35.00 | *-8.73* | Collagen I/II/III/VI/IX/X/XIV, gelatin, ﬁbronectin, aggrecan |
| **MMP14 (MT1-MMP)** | 22.96 | 24.21 | *-1.90* | Collagen I/II/III, gelatin, ﬁbronectin, laminin, entactin, vitronectin, aggrecan |
| MMP15 (MT2-MMP) | 31.16 | 30.53 | 1.94 | Aggrecan, entactin, ﬁbronectin, laminin, tenascin |
| MMP16 (MT3-MMP) | 30.04 | 34.45 | *-16.98* | Collagen III, ﬁbronectin, gelatin |

* Cp = crossing point, Roche software designation for threshold cycle
